# Revealing Subtle Age-Related Balance Differences: Applying Stock Market Indicators to Posturographic Analysis

**DOI:** 10.3390/jcm14238346

**Published:** 2025-11-24

**Authors:** Justyna Michalska, Piotr Wodarski, Jacek Jurkojć, Kajetan J. Słomka

**Affiliations:** 1 Department of Human Motor Behavior, Institute of Sport Sciences, Academy of Physical Education in Katowice, 40-065 Katowice, Poland; 2 Department of Biomechatronics, Faculty of Biomedical Engineering, Silesian University of Technology, 44-100 Gliwice, Poland; piotr.wodarski@polsl.pl (P.W.);

**Keywords:** postural control, trend change index (TCI), center of pressure (COP), tiptoe rising test, Limits of Stability (LOS) test, dynamic balance, age-related balance impairments

## Abstract

**Background/Objectives**: Maintaining postural control is critical for preventing falls, especially in older adults, yet traditional center-of-pressure (COP) analyses may not capture subtle age-related balance impairments. In this study, we integrated a dynamic posturographic assessment—the Tiptoe Rising Test—with an innovative Trend Change Index (TCI) analysis, a method adapted from stock market technical indicators, to enhance the sensitivity of balance evaluations. **Methods**: Twenty-four healthy older adults (65+ years) and twenty healthy young adults (18–30 years) completed both the Limits of Stability (LOS) test and the Tiptoe Rising Test. During each assessment, COP data were continuously recorded via a force plate, and both conventional COP parameters (e.g., sway range, velocity, standard deviation) and dynamic TCI metrics (including TCI_dT, TCI_dS, TCI_dV, and TCI_per_s) were computed. **Results**: Our results indicate that while the LOS test showed limited group differences using standard COP measures—particularly during less dynamic phases—the TCI-derived indices revealed moderate to large effect sizes in capturing temporal and spatial fluctuations in postural adjustments. Notably, the Tiptoe Rising Test, with its inherently dynamic challenge, produced robust differences between young and older participants, with TCI metrics consistently demonstrating enhanced sensitivity in detecting subtle balance impairments. **Conclusions**: These findings suggest that incorporating TCI analysis with dynamic balance tasks, such as the Tiptoe Rising Test, provides a more comprehensive and discriminative assessment of postural control. This integrated approach holds promise for early detection of balance deficits and may inform targeted interventions aimed at fall prevention in the elderly population.

## 1. Introduction

Postural control is a multifaceted process integrating sensory, motor, and cognitive functions to maintain body stability in various conditions. This ability enables individuals to navigate their surroundings safely and efficiently. With advancing age, physiological changes such as decreased muscle strength, impaired proprioception, and delayed reaction times contribute to diminished postural control [1,2]. Early detection of subtle balance deficits is essential, as timely interventions can significantly reduce fall risk and improve quality of life. Falls among older adults remain a major health concern, being a leading cause of injuries, disability, and loss of independence [3,4]. Preventing them is therefore vital for preserving autonomy and reducing healthcare costs.

Accurate assessment of fall risk is crucial for effective prevention. A variety of diagnostic tools are used in clinical and research settings to evaluate equilibrium, strength, and coordination, allowing clinicians to identify individuals at higher risk and tailor interventions accordingly [5]. Common tests include the Limits of Stability (LOS) and the Tiptoe Rising Test, which examine complementary aspects of balance [6,7]. The LOS test measures the ability to maintain stability while reaching in different directions, reflecting the mechanical limits of the functional base of support [8]. In contrast, the Tiptoe Rising Test introduces dynamic postural challenges that engage multiple muscle groups and reveal subtle deficits in stability, making it potentially more sensitive to age-related impairments [9].

In clinical practice, there is an ongoing effort to identify laboratory tests that accurately represent functional tasks encountered in daily life. Consequently, static balance assessments such as the quiet standing test are increasingly being replaced by dynamic evaluations, including the LOS test. Nevertheless, despite its standardized protocol, this test does not fully replicate natural movement patterns observed in everyday activities, as forward weight shifting rarely occurs through isolated ankle motion without hip flexion or heel lift. The Tiptoe Rising Test reflects a real-life activity of reaching for an object overhead. Toe-rise also mirrors the heel-off and forefoot rollover phase of gait, in which body weight is transferred from the heel onto the forefoot before push-off. During the push-off phase of walking, dorsiflexion at the metatarsophalangeal (MTP) joints enables the foot to roll through the toes and generate forward propulsion [10,11]. It complements existing assessments by evaluating an individual’s ability to rise onto the toes, an action essential for tasks such as walking and stair climbing [12]. By assessing the involvement of the calf muscles, the test provides insight into dynamic stability and proprioceptive control [13]. Moreover, the Tiptoe Rising Test may capture aspects of postural control not fully revealed by the LOS test, as it examines balance and stability in a more functional context, demonstrating an individual’s capacity to adapt to the varying demands of everyday movement.

Traditional COP analyses, based on parameters such as standard deviation, velocity, and sway range, provide valuable quantitative insights but may not fully capture the temporal dynamics of balance control. The Trend Change Index (TCI) is an innovative analytical method applied to the COP displacement signal, derived from techniques used in stock market technical analysis. The purpose of TCI is to detect significant changes in the direction of COP movement (i.e., trend changes) while simultaneously filtering out noise and short-term, irrelevant fluctuations [14]. This capability allows TCI to detect both cyclical and non-cyclical (chaotic) components of postural corrections, which traditional frequency domain analyses (like FFT) often ignore. In contrast to nonlinear entropy-based measures such as sample entropy (SampEn), which quantifies the overall regularity or complexity of the COP signal, TCI directly captures the number and timing of meaningful directional changes (reflecting actual postural adjustments) rather than global signal irregularity [15]. Operating in the time domain, the TCI algorithm is less susceptible to noise effects such as spectral leakage commonly encountered in FFT analysis. There is also evidence that the coefficients of variation (CVs) for TCI are much lower, ranging from 1.9% to 10.7%. This low data dispersion suggests that TCI provides significantly more reliable and stable results when analyzing rapid COP movements compared to PSD analysis [16]. The trend change analysis generates a few key parameters used to describe the nature of COP movement between detected postural corrections [17]. These additional indices enhance the understanding of postural dynamics by quantifying specific aspects of COP behavior, such as TCI_dT [s], which measures the time intervals between trend changes; TCI_dS [mm], representing the distances covered during these shifts; and TCI_dV [mm/s], indicating the velocity of movement. By employing TCI analysis, researchers can effectively distinguish between meaningful fluctuations in postural control and random noise that often remain hidden in standard COP measures.

The integration of TCI analysis with both static and dynamic balance assessments may improve early detection of postural instability and support the development of more effective fall-prevention strategies in clinical practice. We expect that in older adults, a greater number and velocity of corrections will be observed during the phase of maintaining a forward body lean as well as during the phase of sustaining a tiptoe stance. This approach is expected to better differentiate postural control capabilities between younger and older adults and to reveal compensatory strategies that remain undetected by conventional COP analysis. Additionally, older adults will exhibit a reduced range of maximum forward lean, and the toe elevation movement will be executed at a slower pace.

## 2. Materials and Methods

### 2.1. Participants

Twenty-four healthy older adults (aged 60–70; *n* = 24) and twenty healthy young adults (aged 18–30; *n* = 20) participated in the study (Table 1). Participants were recruited from the local community. For the older adult group, inclusion criteria were (a) age 60 years or older, (b) no history of falls in the last year, (c) no self-reported cognitive impairments, and (d) the ability to stand independently for at least 30 s. For the young adult group, inclusion criteria were (a) age between 18 and 30 years, (b) absence of any balance impairments or conditions affecting postural stability, and (c) no experience in competitive sports. Exclusion criteria for both groups included any self-reported or clinically diagnosed neurological, musculoskeletal, or vestibular impairments, as well as any condition (e.g., uncorrected severe visual deficits or recent lower limb injuries) that might compromise performance during the balance assessments. All participants provided written informed consent prior to participation, and the study protocol was approved by the Institutional Bioethics Committee of the Academy of Physical Education (approval number: 1/2021). Table 1 presents the anthropomorphic characteristics of young and older adults.

### 2.2. Procedures

**Figure 1:**
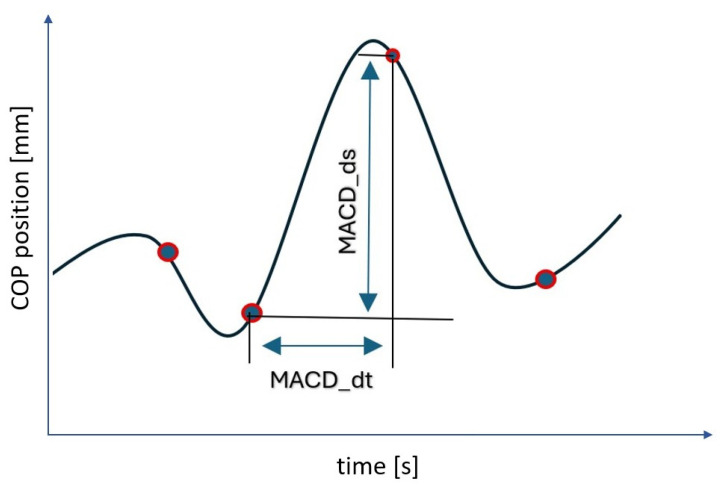
COP displacement signal with marked selected values calculated for two subsequent values of trend change points detected by the algorithm. Legend: MACD_dt—time value between subsequently detected points of a trend change in the COP course; MACD_ds—displacement between subsequent points of the trend change; blue dots—the blue dots correspond to the locations on the COP trajectory where a trend change was detected.

Participants completed two distinct posturographic assessments: the LOS test and the Tiptoe Rising Test. In the LOS test, participants were instructed to lean maximally in the forward direction without lifting their feet or losing balance. The test consisted of three phases: (1) quiet standing for 10 s, (2) forward leaning, and (3) maintaining the inclined position. The data were recorded with the use of a force plate (AccuGait, AMTI) with a sampling rate of 100 Hz. The first step of data processing included the low-pass filtering of each measurement using a 4th-order Butterworth filter with a cut-off frequency of 7 Hz. Force platform data were low-pass-filtered using a 4th-order zero-lag Butterworth filter with a 7 Hz cut-off frequency. This filter was selected to minimize measurement noise while preserving the relevant biomechanical signal, as the dominant frequency components of center-of-pressure movements during quiet stance occur below approximately 5 Hz. The chosen parameters are consistent with previous recommendations for posturographic signal processing [18,19,20]. The next step involved calculating the COP from the ground reaction forces (Fx, Fy, Fz) and moments (Mx, My, Mz). Additionally, we used the algorithm that enables the calculation of the Moving Average Convergence/Divergence (MACD) index to calculate the correction among COP signals. The MACD line is obtained by subtracting two exponential moving averages (EMAs) of lengths 12 and 26 samples. A Signal line was then computed as a 9-sample EMA of the MACD line. These parameters were selected based on the literature to optimize trend-change detection. Points where the MACD and signal lines intersect mark trend changes, and their count defines the Trend Change Index (TCI). Next, time intervals between successive trend-change points form the vector MACD_dT, whose mean is TCI_dT. Displacements between these points yield MACD_dS, with its mean defining TCI_dS. The ratio of corresponding values in MACD_dS and MACD_dT produces MACD_dV, and its mean gives TCI_dV. All indicators can be calculated separately for AP and ML directions (and AP, ML, VT for COM). Final resultant values were obtained by summing directional TCIs and the vector-summed TCI_dT, TCI_dS, and TCI_dV. Earlier publications used the names MACD_dT_mean, MACD_dS_mean, and MACD_dV_mean, corresponding to the same indices (Figure 1).

The maximal voluntary COP excursion (MVE) was defined as the distance between the average COP position in the quiet standing phase and the forward leaning phase. In the Tiptoe Rising Test, participants performed a controlled rising onto their toes. The test consisted of three phases: (1) quiet standing for 10s, (2) rising onto toes, and (3) maintaining the tiptoe position until the end of the trial. All trials lasted 30 s and were repeated three times. Similar to the LOS test, COP data were continuously recorded and analyzed. In both the LOS and Tiptoe Rising Test, subjects were instructed to perform the movement as quickly and as far/high as possible. The duration of the third phase was determined by the velocity of the body when it ended on the velocity of the body leaning or standing on tiptoes. Participants underwent a familiarization session before data collection, and the order of test administration was counterbalanced to control for potential order effects. The results included an analysis of variables, which were examined separately for each phase in the anterior–posterior plane. For Phases I and III, the following parameters were calculated: stdCOP [cm] (standard deviation of COP position), vCOP [cm/s] (velocity of COP), TCI [a.u.] (total number of trend changes during the whole test), TCI_per_s [a.u./s] (trend change index per second), TCI_dS [mm] (mean displacement between trend changes), TCI_dT [s] (mean time between trend changes), and TCI_dV [mm/s] (mean velocity between trend changes [16]. For Phase II, R1 [cm] (LOS range from the mean COP position) and B2 (regression line coefficient indicating forward leaning speed) were calculated [8,17].

Additionally, the subjects’ feet were measured using a caliper, specifically the length of the foot, big toe, and midfoot. The midfoot was defined as the distance between the medial malleolus of the tibia and the metatarsophalangeal joint, while the big toe was defined as the distance between the metatarsophalangeal joint and the tip of the distal phalanx. This allowed for the calculation of the safety margin (SM) for the LOS and Tiptoe Rising Test. SM in postural control refers to the stability reserve that the body maintains during movement to prevent loss of balance and falling [18]. During the LOS test, a person leans forward as far as possible without lifting their heels off the ground. The safety margin is the difference between the maximum displacement of the COP and the boundary of the base of support. The functional base of support during the LOS test is the area represented by the length of the midfoot, measured from the ankle joint to the head of the first metatarsal bone [10]. Naturally, in the Tiptoe Rising Test, the SM is measured relative to the length of the big toe, since it becomes the main point of support. The subjects’ SM is presented in Table 1.

The TCI metrics offer extra information about postural stability by quantifying fluctuations in COP movement. TCI[j]/s presents the number of trend changes per second, whereas TCI_dT [s] represents the mean time interval between successive trend changes, reflecting the temporal regularity of postural adjustments. A shorter TCI_dT [s] suggests frequent corrective movements, potentially indicating instability. TCI_dS [mm] measures the average displacement between successive trend changes, providing insight into the magnitude of postural adjustments. Larger TCI_dS [mm] values may be associated with more pronounced balance corrections. Lastly, TCI_dV [mm/s] represents the mean velocity between trend changes, offering an integrated measure of both time and displacement, and is useful in distinguishing between smooth and abrupt balance corrections [14,16]. All calculations and data analyses were conducted using MATLAB R2024b (Mathworks, Natick, MA, USA).

### 2.3. Statistical Methods

A sensitivity analysis conducted in GPower showed that, given our sample size (*n* = 44), the study had 80% power to detect between-group effects of g ≥ 0.66 at α = 0.05. Therefore, effects smaller than g = 0.66 may not have been reliably detectable.

Data were analyzed using a combination of parametric and nonparametric procedures. First, the distribution of each variable was examined using the Shapiro–Wilk test to assess normality. In cases where the assumption of normality was met, homogeneity of variances was further evaluated using the Brown–Forsythe test. For variables with normally distributed data and equal variances, group comparisons (young vs. older adults) were performed using independent-sample *t*-tests. When the data deviated from normality or the assumption of homogeneity of variances was violated, group differences were tested using the nonparametric Mann–Whitney U test with continuity correction. In addition to *p*-values, effect sizes (ESs) were computed to estimate the magnitude of the observed differences. For parametric tests, Cohen’s d was calculated. For nonparametric tests, the rank-biserial correlation coefficient was used. For parametric analyses using Cohen’s d, an effect size of approximately 0.20 is considered small, around 0.50 is medium, and 0.80 or greater is regarded as large. For nonparametric comparisons, where the effect size is expressed as the rank-biserial correlation coefficient, values of approximately 0.10 indicate a small effect, around 0.30 a medium effect, and 0.50 or greater a large effect. Additionally, following the reviewers’ recommendations, ANCOVA models including height and big toe length as covariates were performed for all COP and TCI variables across all phases of both tests to verify whether anthropometric factors influenced group differences. All statistical analyses were conducted using MATLAB R2022a Mathworks, Natick, MA, USA) and Statistica 13. Figures were generated using JASP (Version 0.19.3, JASP Team, 2025, The Netherlands), an open source statistical software.

## 3. Results

For the LOS test and Tiptoe Rising Test, analyses were conducted separately across three phases. In Phase I, no significant group differences were observed for standard COP measures (stdCOP [cm] and vCOP [cm/s]) and almost all TCI variables (*p* > 0.05), except for TCI_dV [mm/s]. Young subjects were characterized by greater mean velocity between trend changes compared to older subjects (*p* < 0.05) (Table 2).

In Phase II, significant group differences emerged. Young adults leaned further than older subjects (*p* < 0.05). Additionally, young subjects obtained significantly higher values for B2, indicating a more dynamic forward lean movement compared to older subjects (*p* < 0.05) (Table 2).

In Phase III, while standard COP measures remained non-significant, the TCI analysis revealed that TCI variables differed between groups. Young adults obtained significantly higher values for TCI [j], TCI per s [j], and TCI_dV [mm/s] (*p* < 0.05), compared to older adults (Table 2).

**Figure 2:**
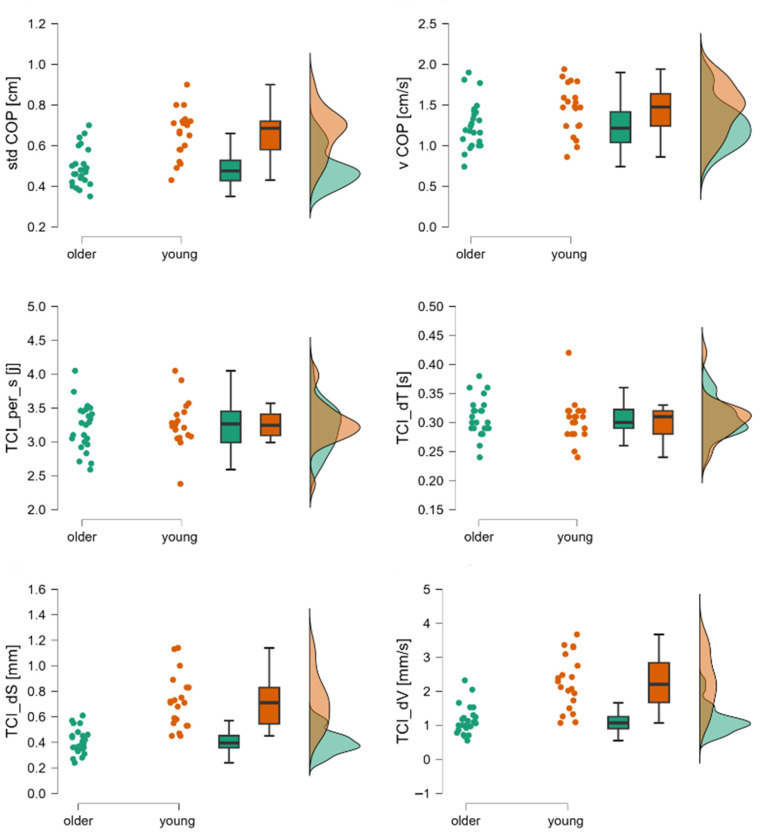
Raincloud plots for stdCOP, vCOP, TCI per_s, TCI_dT, TCI_dS, and TCI_dV. The dot plot represents individual subjects, the box plot shows mean values and confidence intervals, and the distributions correspond to registered data from young and older subjects during the first phase of the Tiptoe Rising Test, corresponding to quiet standing. Legend: stdCOP—standard deviation of COP, TCI_per_s[j]—trend change index per second, TCI_dS—mean displacement between trend changes, TCI_dT—mean time between trend changes, TCI_dV—mean velocity between trend changes, vCOP—velocity of COP.

For the Tiptoe Rising Test, Phase I results indicated robust group differences. The stdCOP [cm], vCOP [cm/s], TCI_dS [mm], TCI_dT [s], and TCI_dV [cm/s] variables were significantly higher in young adults than in older adults (*p* < 0.05), except for the TCI [j] variable, whose value was significantly lower among young adults compared to older adults (*p* < 0.05) (Table 3) (Figure 2).

**Figure 3:**
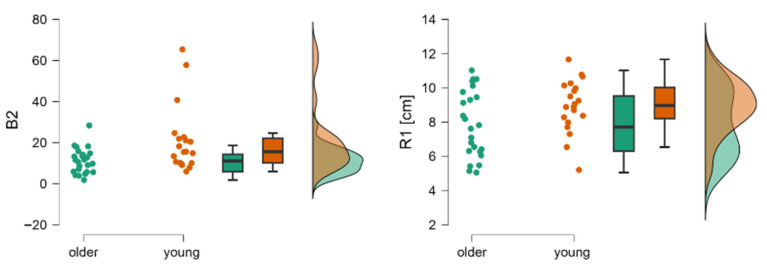
Raincloud plots for B2 and R1. The dot plots represent individual subjects, the box plots show mean values and confidence intervals, and the distributions correspond to data collected from young and older subjects during the second phase of the Tiptoe Rising Test, corresponding to rising onto the toes. Legend: B2—regression line coefficient for Phase II limit of stability test, indicating the speed of leaning forward, R1—limit of stability range calculated from the mean COP position.

In Phase II of the Tiptoe Rising Test, significant group differences emerged. Young adults performed toe-rise movements more quickly (B2) and to a greater height (R1 [cm]) (*p* < 0.05) (Table 3) (Figure 3).

**Figure 4:**
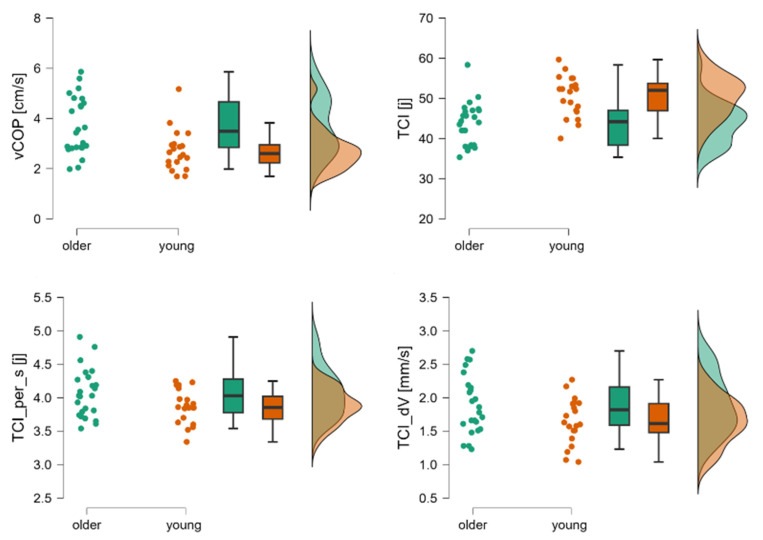
Raincloud plots for vCOP, TCI, TCI per_s, and TCI_dV. The dot plot represents individual subjects, the box plot shows mean values and confidence intervals, and the distributions correspond to registered data from young and older subjects during the third phase of the Tiptoe Rising Test, corresponding to quiet standing. Legend: TCI [j]—total number of trend changes during the whole test, TCI_per_s[j]—trend change index per second, TCI_dV—mean velocity between trend changes, vCOP—velocity of COP.

In Phase III, young adults were characterized by lower values of vCOP [cm/s], TCI per s [j], and TCI_dV [mm/s] compared to older adults. However, the values of TCI [J] were significantly higher among young adults compared to older adults (*p* < 0.05) (Table 3) (Figure 4).

To quantify differences in sensitivity between COP and TCI parameters, we computed mean effect sizes for all variables within equivalent task conditions (LOS and Tiptoe) and phases (1 and 3). Cohen’s d was used as a standardized measure of effect magnitude for all variables, including those originally analyzed with nonparametric tests; rank-biserial correlations (r_pb) were additionally calculated for completeness. Table 4 summarizes the mean effect sizes for COP and TCI variables. In LOS Phase I, TCI demonstrated slightly larger average effects compared with COP (Δd = 0.030). In LOS Phase III, this pattern became more pronounced, with TCI again showing higher sensitivity (Δd = 0.069). A substantial difference between methods was observed in Tiptoe Phase I, where TCI exhibited markedly larger average effect sizes than COP (Δd = 0.588). In Tiptoe Phase III, COP produced larger effects than TCI (Δd = −0.579). Taken together, these results show phase-specific differences in the magnitude of group effects and indicate that TCI, particularly in dynamic conditions (e.g., Tiptoe Phase I), tends to capture stronger between-group contrasts than conventional COP metrics.

Supplementary ANCOVA analyses were performed to evaluate the potential influence of anthropometric factors by including height and big toe length as covariates. The results showed that big toe length did not significantly contribute to any LOS outcomes and affected only two variables in Tiptoe Phase III (TCI_dS and TCI_dV). Height showed isolated effects on COP variables during quiet standing (e.g., stdCOP and vCOP), but did not influence the key dynamic variables of either task. Importantly, all age-related differences in the Tiptoe Rising Test remained significant after adjustment, particularly in the TCI indicators (e.g., TCI[j]AP: *p* = 0.002; TCI_dV: *p* = 0.008). In contrast, the LOS test continued to show limited sensitivity and no meaningful between-group differences after adjustment. Complete ANCOVA results are provided in Supplementary Tables S1 and S2.

## 4. Discussion

A novel aspect of our study was the integration of cross-disciplinary methods to examine postural control. Specifically, we adapted technical analysis techniques originally developed for stock market data—such as the TCI—to analyze posturography signals [14,21]. Whereas conventional COP analysis focuses on parameters like sway range, vCOP, and stdCOP, the TCI approach enables the detection of subtle, dynamic trend changes in the COP trajectory. This method quantifies not only the frequency of postural corrections but also provides detailed indices of the time intervals (TCI_dT [s]), displacements (TCI_dS [mm]), and velocities (TCI_dV [mm/s]) associated with each correction. By borrowing these techniques from financial analysis, our approach offers additional insights into the underlying mechanisms of balance control, thereby enhancing the sensitivity and specificity of posturographic assessments, particularly in distinguishing between different age groups or clinical populations.

As the population ages, maintaining balance becomes increasingly critical for preventing falls and ensuring overall mobility [22]; thus, there is a pressing need for innovative methodologies that can accurately assess and monitor balance performance in older adults. This study set out to determine whether the Tiptoe Rising Test, when combined with TCI analysis, provides a more sensitive assessment of postural control, especially in distinguishing between young and older adults, compared to traditional methods such as the LOS test with standard COP measures. Our findings offer compelling support for the hypothesis that integrating a dynamic, functionally challenging test (the Tiptoe Rising Test) with TCI metrics uncovers subtle balance impairments that conventional COP analyses may overlook. Additionally, the Tiptoe Rising Test is not commonly used, and when it is, typically applied among athletes or younger individuals [23]. Although the TCI parameters (TCI_dT, TCI_dS, TCI_dV) are mathematically well-defined and have already shown potential in differentiating balance performance between groups, the precise physiological meaning of these indices still requires elaboration.

By analyzing quiet standing before performing a motor task (whether it is forward body leaning or rising onto the toes), it can be observed how our verbal command alone influences postural control. Before forward leaning, there were no differences between young and older adults in postural sway as assessed by stdCOP and vCOP. According to the literature [24], there is a trend toward greater postural sway in older adults, but it is not statistically significant. Surprisingly, the initial phase of the Tiptoe Rising Test reveals not only a difference between the groups but also a shift in the trend. On the one hand, older adults exhibited reduced overall postural sway, yet they performed a greater number of corrective adjustments. These corrections were characterized by shorter displacement, lower velocity, and shorter duration compared to those observed in younger individuals. (Figure 2). An increased need to initiate corrective actions indicates an alteration in the mechanisms underlying postural control. This may result from two factors: the previously mentioned difference in the motor task and the overall difficulty of the task. Considering that the anterior functional limit of stability is defined by the line connecting the heads of the first metatarsal bones [8], participants shifted their COP toward this boundary during the LOS test, whereas during the tiptoe test, the COP was displaced beyond this limit.

The third phase of the LOS test involves maintaining the maximal forward-leaning posture, during which COP is displaced as close as possible to the anterior boundary of stability. Younger adults shifted their COP significantly farther than older adults, meaning that the COP of YA was positioned closer to the anterior limit of stability. Nevertheless, both groups exhibited the same level of postural sway. In contrast, older individuals significantly reduced the number of corrections. In the Tiptoe Rising Test, older adults displayed greater postural sway in the toe-standing position, a higher number of corrections per second, and greater correction velocity (Figure 4). When standing on their toes, older adults shifted their COP (see Table 1) closer to the front limit of stability, which led to the initiation of postural recovery strategies as an adaptive response.

TCI_dV (velocity domain) captures the dynamics of trend changes in COP velocity, thus reflecting the responsiveness of the postural control system. Higher TCI_dV values do not necessarily imply maladaptive control. Depending on the context, they may reflect a more responsive, compensatory strategy, especially under conditions of external perturbation [16,17]. However, persistently elevated TCI_dV in static conditions might indicate excessive corrective effort or reduced postural efficiency [25,26]. TCI_dV should be analyzed alongside its kinematic components: TCI_dS and TCI_dT.

The Tiptoe Rising Test, complemented by TCI analysis, proved more sensitive in detecting changes in the mechanism of postural control than traditional LOS tests. The tiptoe task imposes a dynamic challenge requiring rapid, coordinated muscular responses to maintain stability during transitions [27]. This demand appears to accentuate differences between young and older adults, as reflected by the large effect sizes observed in key COP and TCI metrics. In contrast, the LOS test, while valuable, often yielded non-significant differences in its static or less dynamic phases. TCI-derived measures provided a more nuanced view of adjustments by capturing the timing and magnitude of corrective movements. Even in phases where standard measures did not indicate significant group differences, TCI metrics revealed moderate to large effects. This finding underscores the utility of incorporating dynamic indices into postural control assessments, particularly when subtle balance deficits are suspected.

Clinical research supports the relationship between changes in postural control and increased fall risk. Studies using computerized dynamic posturography (CDP) have shown that it is sensitive to changes in postural control, potentially allowing early detection of balance impairments in various populations. For example, Ferrazzoli et al. [28] reported altered sway parameters in Parkinson’s disease patients, while Whitney et al. [29] found that individuals with vestibular disorders and a history of falls had lower equilibrium scores on the Sensory Organization Test. Similarly, Mockford et al. [24] showed that CDP performance worsened with increasing claudication severity. Together, these findings illustrate that CDP effectively detects both central and peripheral balance deficits.

The enhanced sensitivity of the Tiptoe Rising Test and TCI analysis has important clinical ramifications. By identifying subtle changes in dynamic postural control, clinicians can better screen older adults at risk of falls. Early intervention strategies can then be implemented to strengthen balance and prevent falls, potentially reducing healthcare costs and improving quality of life. The detailed insights provided by TCI metrics into the temporal and spatial aspects of balance adjustments can inform targeted therapeutic interventions. For example, rehabilitation programs can focus on improving the speed and magnitude of postural corrections, addressing the specific deficits uncovered by TCI analysis.

In the context of healthy aging, our findings demonstrate that tasks such as tiptoe rising amplify age-related differences in balance, aligning with prior research [30,31], suggesting that dynamic, multi-muscle tasks reveal deficits not evident in static conditions. This approach mirrors contemporary studies on anticipatory postural adjustments (APAs) and multi-muscle synergies [32], particularly within frameworks such as the uncontrolled manifold hypothesis, where COP control reflects neural and biomechanical integration. Integrating novel indices like the TCI into dynamic posturography may therefore enhance early detection of balance impairments and guide the development of targeted interventions. Clinically, identifying subtle deficits in dynamic control can improve screening for fall risk, support early intervention, and inform individualized rehabilitation strategies focused on the speed and magnitude of postural corrections [26].

This study’s limitations include a small, geographically limited sample that may affect generalizability. Additionally, the moderate statistical power may increase the risk of committing a Type II error. However, as highlighted in other data-limited experimental contexts, methodological design and careful feature selection can mitigate low-N biases and enhance generalizability [33]. Future research should use larger, more diverse populations. Although the Tiptoe Rising Test with TCI analysis proved sensitive, its effectiveness in detecting balance deficits in other clinical groups (e.g., neurological patients) remains unclear, so additional functional tasks should be evaluated. Moreover, combining TCI with other diagnostic tools—such as traditional COP measures, EMG, or wearable sensors—may enhance overall diagnostic accuracy and provide a more complete picture of postural control. Future studies should also focus on standardizing TCI metrics and exploring longitudinal designs to better track changes in balance over time.

## 5. Conclusions

In summary, our findings indicate that integrating the TCI with dynamic posturographic assessments—specifically the Tiptoe Rising Test—provides more information about the detection of subtle age-related balance changes than traditional COP metrics alone. Although standard COP measures in LOS test sometimes failed to discriminate between young and older adults, the TCI-derived indices consistently revealed moderate to large effect sizes that underscored significant differences in the temporal and spatial dynamics of postural adjustments. Notably, the Tiptoe Rising Test elicited robust group differences, suggesting that its dynamic nature effectively challenges postural control systems and uncovers subtle deficits that static or less demanding tasks may overlook. These results support the potential clinical utility of this stock market-inspired analytic approach for early identification of balance impairments, which is critical for fall prevention in the elderly.

## Figures and Tables

**Table 1:** Demographic data for all participants.

	Young Adults (*n* = 20)	Older Adults (*n* = 24)
age [years]	20 ± 2	65 ± 5 *
height [cm]	174.55 ± 10.90	163.17 ± 6.60 *
weight [kg]	70.83 ± 19.91	71.86 ± 11.40
foot lenght [cm]	24.50 ± 1.79	25 ± 1.45
metatarsal length [cm]	13.26 ± 1.63	12.61 ± 0.80
big toe length [cm]	5.2 ± 0.7	6.98 ± 0.77 *
stability margin of LOS [%]	12.39 ± 12.55	18.20 ± 15.08
stability margin of Tiptoe Rising [%]	93.17 ± 10.26	90.7 ± 8.72

Legend: * significant differences between groups *p* < 0.05.

**Table 2:** Intragroup comparison between young and older subjects during the LOS test.

Variable	Phase	Young Adults M ± SD Mdn (Min–Max)	Older Adults M ± SD Mdn (Min–Max)	T/Z *p* Value	Cohen’s d /r_pb
stdCOP [cm]	1st	0.47 (0.19–0.77)	0.45 (0.26–0.89)	Z = 0.72; *p* = 0.48	0.13
vCOP [cm/s]		1.15 (0.52–1.69)	1.28 (0.72–2.30)	Z = −1.24; *p* = 0.22	0.23
TCI [j]		34.67 (31.33–45.33)	35.33 (26–43.33)	Z = −0.45; *p* = 0.65	0.08
TCI per s [j]		36.6 ± 0.27	3.27 ± 0.40	t = 0.85; *p* = 0.40	−0.26
TCI dS [mm]		0.42 ± 0.18	0.37 ± 0.12	t = 1.18; *p* > 0.24	−0.35
TCI dT [s]		0.29 ± 0.02	0.3 ± 0.04	t = −1.15; *p* > 0.26	0.32
TCI dV [cm/s]		1.3 ± 0.61	0.96 ± 0.32	**t = 2.35; *p* < 0.02**	**−0.79**
R1 [cm]	2nd	7.51 ± 1.83	5.61 ± 184	**t = 3.20; *p* < 0.00**	**−0.96**
B2		16.39 (4.65–33.66)	7.53 (2.70–24.83)	**Z = 3.43; *p* < 0.00**	**−1.04**
stdCOP [cm]	3rd	0.56 (0.36–0.86)	0.57 (0.28–1.31)	Z = −0.39; *p* = 0.69	0.07
vCOP [cm/s]		1.70 (0.90–2.58)	1.57 (0.81–3.86)	Z = 0.44; *p* = 0.65	0.07
TCI [j]		48.08 (40–66.33)	44.67 (35–57.67)	**Z = 2.49; *p* < 0.01**	**0.44**
TCI per s [j]		4.02 ± 0.59	3.68 ± 0.40	**t = 2.25; *p* < 0.05**	**−0.68**
TCI dS [mm]		0.4 ± 0.11	0.40 ± 0.14	t = −1.98; *p* = 0.16	−0.02
TCI d T [s]		0.25 ± 0.04	0.27 ± 0.03	t = 0.05; *p* = 0.06	0.06
TCI dV [cm/s]		1.26 ± 0.32	1.22 ±0.40	**t = 0.38; *p* < 0.05**	**−0.11**

Legend: Data are expressed as mean ± SD or median (min–max), depending on distribution. *p*-values are based on *t*-tests (parametric) or Mann–Whitney U tests (nonparametric), as appropriate. Effect sizes are reported as Cohen’s d for parametric comparisons and rank-biserial correlation (r_pb) for nonparametric ones. stdCOP—standard deviation of COP position, vCOP—velocity of COP, TCI [j]—total number of trend changes during the whole test, TCI_per_s[j]—trend change index per second, TCI_dS—mean displacement between trend changes, TCI_dT—mean time between trend changes, TCI_dV—mean velocity between trend changes, B2—regression line coefficient for Phase II limit of stability test, indicating the speed of leaning forward, R1—limit of stability range calculated from the mean COP position.

**Table 3:** Intragroup comparison between young and older subjects during the Tiptoe Rising Test.

Variable	Phase	Young Adults M ± SD Mdn (Min–Max)	Older Adults M ± SD Mdn (Min–Max)	T/Z *p* Value	Cohen’s d /r_pb
stdCOP [cm]	1st	0.66 ± 012	0.49 ± 0.09	**t = −5.28; *p* < 0.001**	**−1.60**
vCOP [cm/s]		1.45 ± 0.31	1.26 ± 0.29	**t = −2.14; *p* = 0.039**	**−0.65**
TCI [j]		33.77 ± 3.74	38.86 ±5.20	**t = 3.66; *p* <0.001**	**1.11**
TCI per s [j]		3.28 ± 0.35	3.21 ± 0.35	t = −059; *p* = 0.56	−0.18
TCI dS [mm]		0.71 ± 0.21	0.41 ± 0.10	**t −6.44; *p* < 0.001**	**−1.95**
TCI d T [s]		0.31 (0.24–0.42)	0.30 (0.25–0.38)	**Z = −2.34; *p* = 0.002**	**0.07**
TCI dV [cm/s]		2.21 (1.07–3.67)	1.07 (0.55–2.32)	**Z = 4.47; p < 0.001**	**0.80**
R1 [cm]	2nd	8.95 ± 1.54	7.86 ± 1.94	**t = −2.02; *p* = 0.049**	**−0.61**
B2		15.53 (5.94–65.38)	11.05 (1.78–28.35)	**Z = −2.58; *p* < 0.001**	**0.46**
stdCOP [cm]	3rd	0.67 ± 0.14	070 ± 0.15	t = 0.86; *p* = 0.40	0.26
vCOP [cm/s]		2.60 (1.69–5.17)	3.49 (1.98–5.86)	**Z = −2.91; *p* = 0.003**	**0.52**
TCI [j]		50.50 ± 5.60	43.77 ± 5.27	**t = −4.29; *p* < 0.001**	**−1.3**
TCI per s [j]		3.87 ± 0.26	4.07 ± 0.36	**t = 2.06; *p* = 0.05**	**0.62**
TCI dS [mm]		0.54 ± 0.12	0.60 ± 0.15	t = 1.43; *p* = 0.16	0.43
TCI d T [s]		0.26 ± 0.02	0.25 ± 0.02	t = −1.90; *p* = 0.06	−0.58
TCI dV [cm/s]		1.65 ± 0.34	1.89 ± 0.44	**t = 2.04; *p* = 0.05**	**0.62**

Legend: Data are expressed as mean ± SD or median (min–max), depending on distribution. *p*-values are based on *t*-tests (parametric) or Mann–Whitney U tests (nonparametric), as appropriate. Effect sizes are reported as Cohen’s d for parametric comparisons and rank-biserial correlation (r_pb) for nonparametric ones. stdCOP—standard deviation of COP position, vCOP—velocity of COP, TCI [j]—total number of trend changes during the whole test, TCI_per_s[j]—trend change index per second, TCI_dS—mean displacement between trend changes, TCI_dT—mean time between trend changes, TCI_dV—mean velocity between trend changes, B2—regression line coefficient for Phase II limit of stability test, indicating the speed of leaning forward, R1—limit of stability range calculated from the mean COP position.

**Table 4:** Comparison of average effect sizes (Cohen’s d and rank-biserial r_pb) for COP and TCI variables across LOS and Tiptoe tasks in Phases I and III.

Test	Phase	Cohen’s d COP	Cohen’s d TCI	Cohen Δd
LOS	1 st	−0.23	−0.20	+0.03
	2 nd	−0.21	−0.15	+0.07
TIPTOE	1 st	−1.12	−0.53	+0.59
	2 nd	0.62	0.04	−0.579

Legend: Mean effect sizes (Cohen’s d for parametric variables; rank-biserial correlation r_pb for nonparametric variables) were computed separately for COP and TCI metrics within each task condition (LOS, Tiptoe) and phase (1 and 3). Positive Δ values indicate greater sensitivity of TCI metrics compared to COP metrics. The results demonstrate that TCI exhibits higher discriminatory power in several conditions, particularly in the Tiptoe phase 1 task.

## Data Availability

The datasets generated for this study are available upon request to the corresponding author.

## Supplementary Materials

The following supporting information can be downloaded at: https://www.mdpi.com/article/10.3390/jcm14238346/s1, Table S1: Results of ANCOVA for all COP and TCI variables across LOS test with group as fixed factor and height and big toe length as covariates. Table S2: Results of ANCOVA for all COP and TCI variables across Tiptoe test with group as fixed factor and height and big toe length as covariates.

## Abbreviations

The following abbreviations are used in this manuscript:

B2	Regression line coefficient for the Phase II limit of stability test, indicating the speed of leaning forward
COP	Center of foot pressure
LOS	Limit of stability test
R1	Limit of stability range calculated from the mean COP position
stdCOP	Standard deviation of COP position,
TCI	Trend change index
TCI [j]	Total number of trend changes during the whole test
TCI_per_s[j]	Trend change index per second
TCI_dS	Mean displacement between trend changes
TCI_dT	Mean time between trend changes
TCI_dV	Mean velocity between trend changes
vCOP	Velocity of COP
