# Supplementary material for: Revealing Subtle Age-Related Balance Differences: Applying Stock Market Indicators to Posturographic Analysis
Source: J Clin Med. 2025 Nov 24;14(23):8346. doi: 10.3390/jcm14238346 (PMC12693360; doi:10.3390/jcm14238346)
Supplement: Supplementary file 1 [file jcm-14-08346-s001.zip › jcm-3969699-supplementary.pdf]

Table S1. Results of ANCOVA for all COP and TCI variables across LOS test with group as fixed factor and height and big toe length as covariates.

Variable	phase	Effect	Wald	p-value
**stdCOP**	1 st	height	**4.033**	**0.045**
		toe length	0.191	0.662
		groups	0.558	0.455
**vCOP**		height	**4.943**	**0.026**
		toe length	0.528	0.467
		groups	**4.301**	**0.038**
**TCI[j]AP**		height	3.005	0.083
		toe length	0.205	0.651
		groups	0.439	0.508
**TCI_per_s[j]AP**		height	**4.584**	**0.032**
		toe length	0.005	0.946
		groups	1.736	0.188
**TCI_dT[s]AP**		height	3.815	0.051
		toe length	0.024	0.876
		groups	1.543	0.214
**TCI_dS[mm]AP**		height	**4.984**	**0.026**
		toe length	0.063	0.802
		groups	0.158	0.691
**TCI_dV[mm/s]AP**		height	1.639	0.2
		toe length	0.321	0.571
		groups	0.899	0.343
**R1**	2 nd	height	0.65	0.42
		toe length	0.045	0.831
		groups	2.145	0.143
**B2**		height	0.595	0.44
		toe length	0.207	0.649
		groups	1.417	0.234
**stdCOP**	3 rd	height	3.749	0.053
		toe length	0.387	0.534
		groups	1.742	0.187
**vCOP**		height	3.74	0.053
		toe length	0.126	0.722
		groups	0.443	0.505
**TCI[j]AP**		height	0.175	0.675
		toe length	0.118	0.731
		groups	2.631	0.105
**TCI_per_s[j]AP**		height	0.205	0.651
		toe length	0.055	0.814
		groups	1.524	0.217
**TCI_dT[s]AP**		height	0.026	0.871
		toe length	0.002	0.965

		groups	1.068	0.301
TCI_dS[mm]AP		height	**2.799**	**0.094**
		toe length	0.362	0.547
		groups	0.974	0.324
TCI_dV[mm/s]AP		height	3.305	0.069
		toe length	1.254	0.263
		groups	0.447	0.504

Legend:
stdCOP – standard deviation of COP position, vCOP– velocity of COP, TCI [j] – total number of trend changes during the whole test, TCI_per_s[j] – trend change index per second, TCI_dS – mean displacement between trend changes, TCI_dT – mean time between trend changes, TCI_dV – mean velocity between trend changes, B2 – regression line coefficient for phase II limit of stability test, indicating the speed of leaning forward , R1 – limit of stability range calculated from the mean COP position

Table S2. Results of ANCOVA for all COP and TCI variables across Tiptoe test with group as fixed factor and height and big toe length as covariates.

**Variable**	phase	**Effect**	**Wald**	**p-value**
**stdCOP**	1 st	height	2.866	0.09
		toe length	0.557	0.455
		groups	**8.677**	**0.003**
**vCOP**		height	2.878	0.09
		toe length	0.023	0.88
		groups	0.214	0.644
**TCI[j]AP**		height	0.014	0.907
		toe length	0.095	0.758
		groups	**4.482**	**0.034**
**TCI_per_s[j]AP**		height	0.044	0.833
		toe length	0.023	0.879
		groups	0.245	0.62
**TCI_dT[s]AP**		height	0.669	0.413
		toe length	0.351	0.553
		groups	**14.886**	**0.00**
**TCI_dS[mm]AP**		height	0.017	0.898
		toe length	0.034	0.854
		groups	**0.122**	0.727
**TCI_dV[mm/s]AP**		height	1.146	0.284
		toe length	0.321	0.571
		groups	**12.492**	**0.00**
**R1**	2 nd	height	0.004	0.947
		toe length	0.24	0.625
		groups	0.548	0.459
**B2**		height	0.06	0.807
		toe length	2.463	0.117
		groups	0.203	0.652
**stdCOP**	3 rd	height	**7.317**	**0.007**

	toe length	0.00	0.998
	groups	2.139	0.144
vCOP	height	**4.783**	**0.029**
	toe length	0.156	0.693
	groups	**6.286**	**0.012**
TCI[j]AP	height	0.00	0.996
	toe length	1.183	0.277
	groups	**10.016**	**0.002**
TCI_per_s[j]AP	height	0.057	0.811
	toe length	0.958	0.328
	groups	0.296	0.586
TCI_dT[s]AP	height	0.091	0.763
	toe length	2.098	0.147
	groups	0.038	0.845
TCI_dS[mm]AP	height	**7.604**	**0.006**
	toe length	0.831	0.362
	groups	**5.662**	**0.017**
TCI_dV[mm/s]AP	height	**4.746**	**0.029**
	toe length	1.254	0.263
	groups	**6.932**	**0.008**

Legend:

stdCOP – standard deviation of COP position, vCOP– velocity of COP, TCI [j] – total number of trend changes during the whole test, TCI_per_s[j] – trend change index per second, TCI_dS – mean displacement between trend changes, TCI_dT – mean time between trend changes, TCI_dV – mean velocity between trend changes, B2 – regression line coefficient for phase II limit of stability test, indicating the speed of leaning forward , R1 – limit of stability range calculated from the mean COP position
